# The Relations of the Voluntary Hospitals to the Poor-Law Authorities

**Published:** 1909-03-06

**Authors:** 


### THE RELATIONS OF THE VOLUNTARY HOSPITALS TO THE POOR-LAW AUTHORITIES

A Correspondent sends us the following communication :—

For many years past it has been recognised by all who have studied the question that the existing arrangements are eminently unsatisfactory, and give rise, on the one hand, to wasteful overlapping, and, on the other, to the loss of valuable clinical material for medical education and research. It is not generally realised how great is the development which has taken place during the past thirty years in respect of provision made for the sick poor out of the public rates. The Poor-law expenditure upon the sick poor now exceeds four million pounds per annum, while nearly as much, in addition, is expended by County and Borough Councils on hospitals for infectious diseases and asylums for the insane. In most of our large towns the number of beds provided in the infirmaries, infectious diseases hospitals, and asylums far exceeds that in the voluntary hospitals; for instance, in London the beds provided are as follows :—

| In Voluntary Hospitals | In Poor-law Infirmaries | In Metropolitan Asylum Board Hospitals, &c. |
|---|---|---|
| 9,200 beds | 18,000 beds | 17,600 beds |

In other cities, such as Liverpool, Manchester, Birmingham, etc., the proportions are much the same.

It is evident that the association with the Poor Law does not deter the sick poor from utilising the infirmaries, many of which are at the present day model institutions most admirably equipped; but, sad to say, almost entirely unused for the purposes of medical education. The result is that the rising generation of medical practitioners receives clinical instruction in the wards of our voluntary hospitals, where there are only a comparatively small number of the more acute cases, while the chronic and fever cases, with which they will have most to do in private practice, are being treated in these publicly supported institutions, which are not available for educational purposes.

The Commission recommend the appointment by the "Public Assistance Authority" of special Medical Assistance Committees, who shall have power to co-operate with the existing voluntary hospitals, dispensaries, and friendly societies, and with public health and education authorities, such co-operation to be based upon a clear definition of their respective functions.

The majority report advocates that medical assistance be organised upon a provident basis, that a general system of provident dispensaries be established, and every inducement offered to the working classes below a certain wage limit to become members of them. Under our present arrangements the out-patient departments of most of our voluntary hospitals are open free to all applicants for treatment, and the provident dispensaries have great difficulty in securing members; unless some drastic change is made in the direction of rigidly limiting the out-patient departments to such patients as have been recommended by medical practitioners for consultative purposes, there is little prospect of this recommendation becoming more than a mere counsel of perfection. The report urges that whatever reorganisation takes place, due regard should be given to all voluntary aid organisations, and that, to avoid waste and overlapping, a new organisation be established by statute to form a link between "The Public Assistance Authorities" and charitable efforts.

